# Profiles of a Health Information–Seeking Population and the Current Digital Divide: Cross-Sectional Analysis of the 2015-2016 California Health Interview Survey

**DOI:** 10.2196/11931

**Published:** 2019-05-14

**Authors:** Hena Naz Din, Corinne McDaniels-Davidson, Jesse Nodora, Hala Madanat

**Affiliations:** 1 University of California, San Diego La Jolla, CA United States; 2 School of Public Health San Diego State University San Diego, CA United States; 3 Institute for Public Health San Diego State University San Diego, CA United States; 4 Moores Cancer Center University of California, San Diego La Jolla, CA United States; 5 Department of Family Medicine and Public Health School of Medicine University of California, San Diego La Jolla, CA United States; 6 Institute for Behavioral and Community Health San Diego State University San Diego, CA United States

**Keywords:** internet, digital divide, health information, patient portals, online health information seeking, eHealth

## Abstract

**Background:**

Internet use for health information is important, given the rise of electronic health (eHealth) that integrates technology into health care. Despite the perceived widespread use of the internet, a persistent “digital divide” exists in which many individuals have ready access to the internet and others do not. To date, most published reports have compared characteristics of internet users seeking health information vs nonusers. However, there is little understanding of the differences between internet users seeking health information online and users who do not seek such information online. Understanding these differences could enable targeted outreach for health interventions and promotion of eHealth technologies.

**Objective:**

This study aims to assess population-level characteristics associated with different types of internet use, particularly for seeking online health information.

**Methods:**

The 2015-2016 California Health Interview Survey datasets were used for this study. Internet use was classified as never used the internet (*Never use*), ever used the internet but not to search for health information in the last 12 months (*Use not for health*), and ever used the internet and have used it to search for health information in the last 12 months (*Use for health*). Weighted multinomial logistic regression was used to assess sociodemographic and health characteristics associated with types of internet use. Findings are reported as odds ratios (ORs) with 95% CIs.

**Results:**

Among 42,087 participants (weighted sample of 29,236,426), 19% reported *Never Use* of the internet, 27.9% reported *Use not for health*, and 53.1% reported *Use for health*. Compared to *Never Use* individuals, *Use for health* individuals were more likely to be younger (OR: 0.1, 95% CI 0.1-0.2 for ≥60 years vs <60 years), female (OR: 1.6, 95% CI 1.3-1.9 compared to males), and non-Hispanic white (OR: 0.54, 95% CI 0.4-0.7 for Latinos and OR: 0.2, 95% CI 0.2-0.4 for African Americans) and have a higher socioeconomic status (>400% of Federal Poverty Guidelines; OR: 1.3, 95% CI 1.4-2.4). Overall, characteristics for the *Use not for health* and *Use for health* groups were similar, except for those with lower levels of education and respondents not having visited a physician in the last year. For these two characteristics, the *Use not for health* group was more similar to the *Never Use* group.

**Conclusions:**

Our findings indicate that a digital divide characterized by sociodemographic and health information exists across three types of users. Our results are in line with those of previous studies on the divide, specifically with regard to disparities in use and access related to age, race/ethnicity, and socioeconomic status. Disparities in online health-seeking behavior may reflect existing disparities in health care access extending into a new era of health technology. These findings support the need for interventions to target internet access and health literacy among *Never Use* and *Use not for health* groups.

## Introduction

The internet has become a widespread tool of social communication, economic opportunity, and health care information and access [1]. Since 2000, use of the internet has increased nationally from 52% to 89% [2]. In the realm of health and well-being, access to and use of the internet are central to the rise of electronic health (eHealth), which is the integration of technology into health care to improve and facilitate wellness and health maintenance [3-5]. The Affordable Care Act, in particular, has expanded the integration of the internet in health care by funding and incentivizing health information technology in the form of patient portals and electronic health records [6]. Although these advances are intended to improve patient wellness, a digital divide exists in which certain individuals have ready access to and can use the internet, whereas others do not [7]. This divide has the potential to exacerbate existing health disparities among vulnerable populations.

Although the digital divide was historically characterized by access to the internet, more recently, the divide has highlighted differences in use and skills [4]. Use of the internet for health information has real-world benefit, including application of information learned online to manage and monitor one’s health [4,8,9]. However, internet access and use vary by individual characteristics including race and ethnicity. Prior studies report that individuals of older age, lower income, male gender, and rural locality tend to be less likely to report internet access or use compared to their counterparts [7,10]. Latinos, African Americans, and individuals with low English proficiency are more likely to report no internet use compared to non-Hispanic Whites and English-proficient individuals [7,11]. Assessing characteristics of those who do and those who do not use the internet for health information may be important within the context of the digital divide, because it has implications on the potential impact of eHealth. Specifically, groups least likely to report internet use are also less likely to report use of patient portals and access online health information [12,13].

Published reports about internet use, specifically for seeking health information, have primarily compared individuals who do not use the internet in any capacity to those who use the internet specifically for seeking health information [7,10]. This type of dichotomy tends to focus primarily on internet use in the context of access (ie, those who have access to the internet for seeking health information are users and those who do not are nonusers). This overlooks a third group that exists—individuals who use the internet but not to seek health information. To our knowledge, only one other study explored characteristics of these individuals in comparison to internet users who seek health information online [7]. However, characterization of groups by internet use should include nonusers of the internet as well as other types of users. By delineating group characteristics according to the type of use, findings may reveal new opportunities for intervention.

Therefore, this study aims to assess population-level characteristics associated with internet use, particularly for seeking health information online. Sociodemographic and health characteristics will be explored among those who report no use of the internet, those who use the internet for seeking health information, and those who use the internet but not for seeking health information. Varied profiles of use and nonuse may indicate areas of the digital divide that are changing and may benefit from intervention and targeted resources.

## Methods

### Data Source and Participants

Data from the 2015-2016 California Health Interview Survey (CHIS) were used for this study. CHIS is a statewide study conducted by the University of California Los Angeles Center for Health Policy and Research in collaboration with the California Department of Public Health and the Department of Health Care Services. Data are collected annually through surveys administered by random-digit dialing to both landline phones and cellphones. CHIS data yield a representative sample of the noninstitutionalized population of California to explore health, wellness, and access by race and region across the state.

This study used publicly available data files that allow for estimation of state-level outcomes. The 2015-2016 CHIS data files are provided separately for each year and require pooling to aggregate data. The first wave of data was collected from May 2015 to February 2016 and the second, from January 2016 to December 2016. Participants were recruited through landline and cell phone sampling. All individuals who participated in the adult (age≥18 years) 2015-2016 surveys were included in this study.

### Types of Internet Use

The CHIS assesses types of internet use by asking participants “Have you ever used the Internet?” and “In the past 12 months, did you use the Internet to look for health or medical information?” [14]. Responses to questions were “yes” or “no.” Only those who responded “yes” to having used the internet were asked about searching for health or medical information online. Internet use could be on any computer or electronic device and encompassed a variety of activities like emailing and searching the Web. Health and medical information was broadly defined as searching for health and wellness advice (eg, nutrition and physical activity), disease symptoms, health plans, and other related topics [14]. These questions were combined to create the main outcomes of this study: never used the internet (*Never use*), ever used the internet but have not used it to search for health information in the last 12 months (*Use not for health*), and ever used the internet and have used it to search for health information in the last 12 months (*Use for health*).

### Measures

Participant sociodemographic and health information characteristics were included to assess association with the types of internet use. Variables were selected based on previous literature indicating an association of internet access with type of use and online health seeking. Sociodemographic variables included age, gender, marital status, education, primary language used at home, nativity, household size, federal poverty level, and race/ethnicity. Age was categorized as <60 years and ≥60 years based on past research, which highlights a disparity in internet use around this age [10]. Race and ethnicity are reported in CHIS based on the Office of Management and Budget classification and included six categories: Hispanic, Non-Hispanic (NH) white, African-American (NH), Asian (NH), American Indian/Alaskan Native (NH), and Other races. Rural and urban locality is a CHIS-generated variable based on zip code data.

Health information included the type of health insurance (private, public, or no insurance), presence of usual care (“yes” was classified as access to a doctor or clinic/health center and “no” was classified as no reported usual care, emergency department listed as usual care, or no one place used for usual care), current health status (excellent, very good, good, fair, or poor), and visit to a medical provider in the last 12 months (“yes” or “no”).

### Weighting of Data

As recommended by the CHIS, this study utilized weights for replication methods. The CHIS uses a complex sampling design that involves oversampling by geographic area and group characteristics (eg, race/ethnicity). Weights are thus needed to adjust for the different probabilities of selection and to most accurately reflect the population of California. Design characteristics that require weighted variables include multiple people interviewed in one household (maximum 3), oversampling from certain geographic areas, and random-digit phone dialing for participant recruitment. Weights are designed for the jackknife method of variance and bias estimation.

### Statistical Analyses

Descriptive statistics on internet use and variables of interest were performed using design-adjusted or weighted frequencies. Correlations between variables were assessed to avoid multi-collinearity; closely identified variables (rho≤0.5) were reduced to one variable for inclusion in the final model [15]. Bivariate analyses were conducted between each variable and internet use in order to determine inclusion into the final model at a significance of *P*<.05. To evaluate the association between the variables of interest and internet use, weighted multinomial logistic regression analysis was conducted to model internet use outcomes, using the *Never* *Use* group assigned as the reference group. Findings are reported as odds ratios (OR) with 95% CIs. The difference of association between *Use not for health* and *Use for health* groups was assessed by evaluating overlapping CIs from the weighted multinomial logistic regression. Evaluating CIs is a conservative method of assessing significant differences as compared to other post-hoc analyses/comparisons of groups [16,17]. Assessment of CIs focused on CIs that did not overlap, thus indicating a significant difference between groups [16,17]. All analyses were conducted using SAS OnDemand for Academics (SAS Institute Inc, Cary, NC).

## Results

### Study Population

The CHIS sampled 21,034 individuals in 2015 and 21,055 in 2016. This resulted in a population-weighted sample of 14,541,326 from 2015 and 14,695,100 from 2016. The final population-weighted sample comprised 29,236,426 individuals.

### Prevalence of Internet Use

*Never use* of the internet was reported by 83.8% (n=33,856) of the study sample (weighted: 84.2%, n=24,508,603). Among those*,* 65.6% (n=22,195; weighted: 65.4%, n=16,027,194) reported using the internet to search for health information in the last 12 months. In addition, 19% (n=7959, weighted: 15.8%, n=4,599,254) reported *Never Use* of the internet, 27.9% (n=11,641; weighted: 29.1%, n=8,473,075) reported *Use not for health*, and 53.1% (n=22,194; weighted: 55.1%, n=16,026,161) reported *Use for health*.

### Internet Use by Population Characteristics

The distribution of population characteristics across levels of internet use is reported in Table 1. Comparison of proportions showed a significant difference (*P*<.05) within each characteristic across all categories of internet use. A greater percentage of older adults, Hispanics, and individuals with public health insurance were found in the *Never Use* group (Table 1). Public and private health insurance was split similarly among the *Use not for health* group (42.7% vs 45.3%). A majority of the *Never Use* group reported good or fair health (30.3% vs 34.9%), while very good and good health were more common among *Use not for health* (29.4% vs 32.9%) and *Use for health* (34.2 vs 29.8) groups (Table 1).

Weighted regression showed that compared that to *Never Use* individuals, *Use for health* individuals were more likely to be younger (OR: 0.1, 95% CI 0.1-0.2 for ≥60 years vs <60 years), female (OR: 1.6, 95% CI 1.3-1.9 compared to males), and non-Hispanic white (OR: 0.5, 95% CI 0.4-0.7 for Latinos and OR: 0.2, 95% CI 0.2-0.4 for African Americans), and have a higher socioeconomic status (≥400% of Federal Poverty Guidelines; OR: 1.3, 95% CI 1.4-2.4; Table 2).

Living in an urban location was significant for *Use for Health* compared to *Never Use* (OR: 0.7 95% CI 0.6-0.9 for rural vs urban); however, there was no significant difference in geographic locality between *Never Use* and *Use not for health.* *Use for health* individuals were also more likely to be employed (OR: 0.5, 95% CI 0.4-0.7 for unemployed vs employed) and privately insured (OR: 1.7, 95% CI 1.4-2.2 compared to *Never Use*; Table 2).

**Table 1:** Distribution of population characteristics according to type of internet use (weighted population values) from the 2015-2016 California Health Interview Survey.

Characteristic		*Never use* ^a^	*Use not for health* ^a^	*Use for health* ^a^
**Age (years), n (%)**				
	<60	2,057,498 (44.7)	6,640,214 (78.4)	12,907,147 (80.5)
	≥60	2,541,756 (55.3)	1,832,861 (21.6)	3,119,014 (19.5)
**Gender, n (%)**				
	Male	2,137,475 (46.5)	4,845,123 (57.2)	7,223,238 (45.1)
	Female	2,461,779 (53.5)	3,627,952 (42.8)	8,802,923 (54.9)
**Race^b^** **, n (%)**				
	Non-Hispanic white	1,038,704 (22.6)	3,069,101 (36.2)	8,058,396 (50.3)
	Hispanic	2,650,416 (57.6)	3,489,369 (41.2)	4,164,014 (26)
	African American	271,686 (5.9)	628,620 (7.4)	736,405 (4.6)
	American Indian/Alaskan Native	25,902 (0.56)	51,779 (0.61)	56,660 (0.35)
	Asian	561,019 (12.2)	1,037,029 (12.2)	2,548,049 (15.9)
	Other	51,528 (1.1)	197,176 (2.3)	462,638 (2.9)
**Household size**				
	Reponses obtained, n (%)	4,599,254 (15.8)	8,473,075 (29.1)	16,026,161 (55.1)
	Mean (SEM)	3.20 (0.06)	3.43 (0.04)	3.15 (0.02)
**Employment^c^** **, n (%)**				
	Employed	2,837,920 (61.7)	5,748,514 (67.8)	11,358,968 (70.9)
	Unemployed	1,761,334 (38.3)	2,724,560 (32.2)	4,667,193 (29.1)
**% Federal Poverty Level, n (%)**				
	0-138	2,339,046 (50.9)	2,561,345 (30.2)	3,653,178 (22.8)
	139-200	687,038 (14.9)	1,018,756 (12.0)	1,562,263 (9.7)
	201-400	938,716 (20.4)	2,158,134 (25.5)	3,683,057 (23.0)
	≥400	634,454 (13.8)	2,734,840 (32.3)	7,127,664 (44.5)
**Geographic location^d^** **, n (%)**				
	Urban	4,096,249 (89.1)	7,593,284 (89.6)	14,582,763 (91)
	Rural	503,005 (10.9)	879,791 (10.4)	1,443,398 (9.0)
**Place of birth, n (%)**				
	The United States	1,702,387 (37.0)	5,407,011 (63.8)	12,069,324 (75.3)
	Outside the United States	2,896,866 (63)	3,066,064 (36.2)	3,956,837 (24.7)
**General health, n (%)**				
	Excellent	453,312 (9.8)	1,580,799 (18.7)	3,265,295 (20.4)
	Very good	647,263 (14.1)	2,494,385 (29.4)	5,481,351 (34.2)
	Good	1,392,301 (30.3)	2,785,481 (32.9)	4,777,245 (29.8)
	Fair	1,606,978 (34.9)	1,331,452 (15.7)	2,020,028 (12.6)
	Poor	500,499 (10.9)	280,958 (3.3)	482,242 (3.0)
**Insurance type^e^** **, n (%)**				
	Public	3,216,169 (69.9)	3,619,481 (42.7)	5,316,902 (33.2)
	Private/employer	798,882 (17.4)	3,835,796 (45.3)	9,498,872 (59.3)
	Uninsured	584,203 (12.7)	1,017,798 (12.0)	1,210,387 (7.6)
**Education level** **, n (%)**				
	Less than high school	2,440,420 (53.1)	1,575,314 (18.6)	962,512 (6.0)
	High school	1,104,174 (24.0)	2,368,448 (28.0)	2,900,243 (18.1)
	Some college^f^	575,168 (12.5)	2,184,604 (25.8)	4,115,447 (25.7)
	Bachelor of Arts/Science	360,689 (7.8)	1,600,450 (18.9)	5,062,836 (31.6)
	Master’s degree or higher	118,802 (2.6)	744,259 (8.8)	2,985,123 (18.6)
**Usual source of care^g^** **, n (%)**				
	Yes	3,710,563 (80.7)	6,889,486 (81.3)	13,892,613 (86.7)
	No	888,691 (19.3)	1,583,588 (18.7)	2,133,548 (13.3)
**Visited physician in the last 12 months** **, n (%)**				
	Yes	3,673,726 (79.9)	6,125,379 (72.3)	13,663,846 (85.3)
	No	925,528 (20.1)	2,347,696 (27.7)	2,362,315 (14.7)
**Marital status** **, n (%)**				
	Married	2,321,581 (50.5)	3,865,368 (45.6)	7,733,475 (48.3)
	Other^h^	1,715,317 (37.3)	2,078,225 (24.5)	3,377,259 (21.1)
	Never married	562,356 (12.2)	2,529,483 (29.9)	4,915,426 (30.7)
**Language at home** **, n (%)**				
	English	1,464,336 (31.8)	4,388,202 (51.8)	9,998,790 (62.4)
	Spanish	1,405,725 (30.6)	876,315 (10.3)	587,631 (3.7)
	Asian languages^i^	231,906 (5.04)	182,780 (2.2)	267,543 (1.7)
	Other	56,752 (1.2)	126,799 (1.5)	227,592 (1.4)
	Multilingual	1,440,535 (31.3)	2,898,979 (34.2)	4,944,605 (30.9)

^a^*Never use*: never used the internet; *Use not for health*: ever used the internet but not for seeking health information in the last 12 months; *Use for health*: used internet ever and for health information in the last 12 months.

^b^Based on the classification by the Office of Management and Budget [18].

^c^“Employed” includes those with full- and part-time employment; “Unemployed” includes those looking for work and those not looking for work.

^d^Determined by zip codes.

^e^Public: categorized as only Medicare, only Medicaid, combination of the two, and the combination of one with a classified *other* insurance. Private: categorized as employment-based insurance and privately purchased insurance.

^f^“Some college” includes vocational school, Associates of Arts, Associates of Science, and some years of college.

^g^Usual care includes doctor or clinic/health center. No usual care includes no reported usual care, emergency department listed as usual care, or no one place used for usual care.

^h^“Other” includes widowed, separated, divorced, or living with partner.

^i^Asian languages include Chinese, Vietnamese, and Korean.

**Table 2:** Association between population characteristics and type of internet use (weighted population values). Values are presented as multivariable-adjusted odds ratio and 95% CI (Reference-*Never use*).

Characteristic		*Use not for health* ^a^	*Use for health* ^a^
**Age (years)**			
	<60 years	1	1
	≥60 years	0.2 (0.1-0.2)	0.1 (0.1-0.2)
**Gender^b^**			
	Male	1	1
	Female	0.9 (0.8-1.1)	1.6 (1.3-1.9)
**Race **			
	Non-Hispanic white	1	1
	Hispanic	0.79 (0.58-1.06)	0.54 (0.40-0.72)
	African American^b^	0.64 (0.48-0.84)	0.29 (0.21-0.39)
	American Indian/Alaskan Native	0.77 (0.43-1.40)	0.42 (0.24-0.72)
	Asian	0.70 (0.48-1.03)	0.69 (0.46-1.03)
	Other	0.89 (0.51-1.56)	0.79 (0.44-1.40)
Number of individuals in a household, mean (SEM)		1.1 (1.0-1.2)	1.1 (1.0-1.1)

**Employment**			
	Employed	1	1
	Unemployed	0.6 (0.5-0.7)	0.5 (0.4-0.7)
**% Federal Poverty Guidelines**			
	0-138	1	1
	139-200	1.1 (0.8-1.4)	1.0 (0.8-1.3)
	201-400	1.5 (1.1-1.9)	1.3 (1.04-1.7)
	>400	1.9 (1.4-2.4)	1.8 (1.4-2.4)
**Geographic location**			
	Urban	1	1
	Rural	0.9 (0.7-1.1)	0.7 (0.6-0.9)
**Place of birth**			
	The United States	1	1
	Outside the United States	0.6 (0.4-0.7)	0.4 (0.3-0.5)
**General health**			
	Excellent	1	1
	Very good	1.1 (0.8-1.4)	1.1 (0.8-1.5)
	Good	0.9 (0.7-1.2)	0.9 (0.7-1.2)
	Fair	0.6 (0.4-0.9)	0.7 (0.5-1.0)
	Poor	0.5 (0.4-0.7)	0.6 (0.4-0.9)
**Insurance type**			
	Public	1	1
	Private/employer	1.4 (1.1-1.7)	1.7 (1.4-2.2)
	Uninsured	1.1 (0.8-1.5)	1.4 (0.9-1.9)
**Education level**			
	Less than high school	1	1
	High school	2.3 (1.8-3.1)	3.5 (2.6-4.6)
	Some college^b^	3.9 (2.8-5.3)	8.2 (5.9-11.2)
	Bachelor of Arts/Science^b^	4.5 (3.2-6.3)	15.3 (10.8-21.7)
	Master’s degree or higher^b^	7.1 (4.5-11.4)	32.1 (19.6-52.4)
**Usual source of care**			
	Yes	1	1
	No	0.9 (0.7-1.1)	0.9 (0.7-1.2)
**Visited physician in the last 12 months^b^**			
	Yes	1	1
	No	1.2 (1.0-1.6)	0.6 (0.5-0.8)
**Marital status**			
	Married	1	1
	Other	0.9 (0.8-1.1)	0.9 (0.7-1.1)
	Never married	1.6 (1.2-2.1)	1.8 (1.3-2.4)
**Language at home**			
	English	1	1
	Spanish	0.6 (0.4-0.8)	0.5 (0.3-0.7)
	Asian languages	0.8 (0.4-1.7)	0.5 (0.2-1.1)
	Other	0.7 (0.3-1.6)	0.5 (0.2-1.2)
	Multilingual	0.9 (0.7-1.2)	1.0 (0.7-1.3)

^a^*Use not for health*: ever used the internet but not for seeking health information in the last 12 months; *Use for* *health*: used internet ever and for health information in the last 12 months.

^b^Significant difference across *Use not for health* and *Use for* *health* groups assessed by 95% CI overlap.

The *Use not for health* group had similar characteristics as the *Use for health* group, except in the likelihood of being female, likelihood of being African American (non-Hispanic), levels of education, and likelihood of not having visited a physician in the last year. Compared to *Use for health* individuals, *Use not for health* individuals were significantly less likely to be female but significantly more likely to be African American (NH). Compared to *Never Use* individuals, more *Use not for health* individuals had college level or higher education (OR: 4.5, 95% CI 3.2-6.3 for Bachelor of Arts/Bachelor of Science and OR: 7.1, 95% CI 4.5-11.4 for master’s degree or higher). These CIs did not overlap when compared to *Use for health* (OR: 15.3, 95% CI 10.8-21.7 for Bachelor of Arts/Bachelor of Science and OR: 32.1, 95% CI 19.6-52.4 for master’s degree or higher), indicating a larger proportion of people with higher education among the *Use for health* group. Not visiting a physician in the last year was not significantly different between the *Never Use* and *Use not for health* groups (OR: 1.2, 95% CI 1.0-1.6) but was different when compared to the *Use for health* group (OR: 0.6, 95% CI 0.5-0.8).

**Figure 1:**
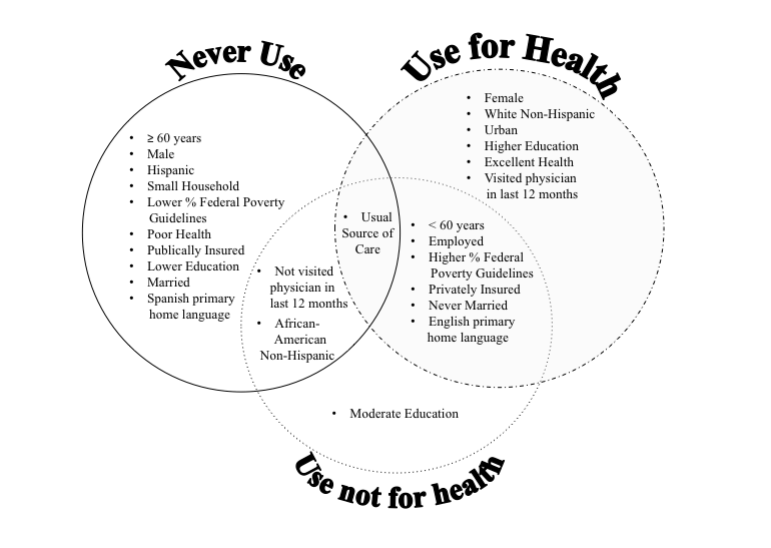
Characteristics of groups by types of internet use. Never use: never used the internet; Use not for health: ever used the internet but not for seeking health information in the last 12 months; Use for health: used internet ever and for health information in the last 12 months.

Overall, the *Use not for health* and *Use for health* groups shared more similarities in sociodemographic characteristics than the *Never Use* group (Figure 1). Only the usual source of care did not significantly differ between the groups.

## Discussion

### Principal Results

This study assessed population characteristics associated with types of internet use for seeking health information among a population representative sample in California. The study adds to the literature by updating the assessment of the digital divide across three categories of use.

### Limitations

Strengths of this study include use of a population representative sample of California and assessment of internet use, specifically for seeking health information online. The size of the sample and subsequent weighting allowed for nuanced evaluation of small but key groups within California like racial minority and rural residing groups. Limitations of the study include lack of specificity in terms of what type of health information respondents sought online and with what frequency respondents sought this information. Additionally, CHIS does not report on other aspects considered important for internet use including health literacy and technical skills to navigate online searching. As a population-based study specific to California, the CHIS results are not generalizable to other states. Additionally, CHIS is based on self-report and may be subject to recall bias.

### Comparison to Prior Work

Compared to the study by Nguyen et al [7], the proportion of Californians who are internet users and who searched for health information has grown from 81.5% to 84.2% and from 64.5% to 65.4%, respectively [7]. This study shows that compared to *Use for health* individuals*, Never Use* individuals tend to be older, male, Hispanic, or African American (vs NH white); be of a lower socioeconomic status; and report poorer health (compared to excellent health). These distinctions mirror what is already understood about the digital divide, specifically with regard to age disparities (older adults vs adults) and racial disparities (African American/Hispanic vs NH white) [7,19]. Although general internet use is growing among older adults (67%) and racial/ethnic minorities (87%), these groups still report less internet use and access [2]. Older adults are a vulnerable group, given their high health needs, and may benefit from internet use for health. Barriers to internet use among older adults include technical difficulties, confusion with the amount of information online, disability in terms of psychomotor function, health literacy, costs, and distrust in internet sources [10,20]. Of note, distrust and low health literacy (eg, understanding health and medical information) have been significantly associated with less internet use for seeking health information even when older adults report using the internet [10,20].

Among African American and Hispanic individuals, internet access, defined by desktop, laptop, or handheld computer (ie, smartphone) ownership or broadband subscription, is low (63.5% and 69.6%, respectively) as compared to NH white individuals (78.8%) [21]. Smartphones have helped increase access to the internet for minority groups. Compared to only 9% of NH white individuals, 22% of Hispanic and 15% of African American individuals are smartphone-only internet users [22]. Among these groups, activities such as health information seeking are more likely to be performed on smartphones than on a traditional computer. Among Hispanic individuals, nonusers tend to be less proficient in English, and Spanish is the primary language in which media is consumed [11]. Language and literacy barriers are significant, as searching, reading, and comprehending health information online can be complex. Use of a patient portal may be more complicated than searching for health information online, as the former involves logging in, password creation, and more specific medical information. Thus, language and literacy are significant barriers to internet use generally and in the context of eHealth.

Unique to our study is the classification of *Use not for health* and comparing this group to both *Never Use* and *Use for health* groups. Given the saturation of internet use in our sample (>80% reported internet use), we were able to subcategorize this group into use but not for health and use for health seeking. Differences in educational attainment, gender, race (specifically of African American race), and physician visits in the last year differentiate the *Use not for health* group from the *Use for health* group. Nguyen et al [7] reported similar findings when only characterizing internet users who search for health information online. Specifically, they found that individuals who use the internet to search for health information were more likely to have seen a physician in the past year, to have higher educational attainment, to be of an ethnic minority (ie, African American or Latino), and to be female [7]. Not having visited a physician in the last 12 months may explain the low use of the internet for seeking health information, because it may indicate no serious diagnosis and thus no concerns or interests in seeking health information online. A serious diagnosis, or risk of one, is associated with health information seeking, as evidenced by a national US sample in which health information seeking was more prevalent among cancer survivors (69.8%) and individuals with a family history of cancer (51.2%) than those who had no history of cancer (29.6%) [23]. In the context of eHealth and health technology integration, it is important to explore why individuals who have access to the internet in some capacity do not use it for health-seeking activities (eg, searching for health information).

Types of internet use may indicate the extent to which eHealth expansion and integrated online health tools may be missing key populations. Health systems and policy makers may consider the characteristics of populations identified in this study by *Never Use* and *Use not for health* groups, because these populations may be lacking in the intended benefit of patient portals and eHealth resources. In particular, if *Never Use* and *Use not for health* groups do not frequent medical providers, accessing these groups may require other techniques besides, for example, designating a patient portal during a clinic visit. Types of use may also reflect existing disparities in health care access continuing into a new era of health technology. As evidenced by a study of a representative sample in the Dutch population, disparities in internet usage reflected real-life disparities experienced within the Dutch population according to gender, age, and level of education [24]. Careful consideration and purposeful design of health and technology integration may have the potential to eliminate disparities [25]. Patient portals have already been shown to improve patient outcomes and engagement and reduce health care costs, especially among patients with chronic conditions [26]. However, this only applies to patients who are able to access, use, and comprehend the information being shared through these portals. Healthy People 2020 has incorporated internet access and use into its objectives to target these disparities [27]. Particularly relevant are objectives to increase the proportion of online health information seekers who report easy access to information to increase health literacy and the proportion of health-related websites that are simple and usable [27].

### Conclusions

Advances in health care and management have promoted internet integration and its use to support health and wellness. Although our study found a high prevalence of internet use across the study population, findings suggest that a digital divide continues to exist. The widest divide still remains between nonusers and users, and a lesser divide exists among users who search for health information and users who do not. Disparities identified in both internet use and health information seeking reflect a lack of health equity in a new era of technological advances in society and health care. The internet is one important tool for the development of an empowered patient and individual. Underutilization of the internet as a tool of health information leaves behind vulnerable populations and may have an adverse impact on health care for these individuals. Future studies may explore specific barriers to internet use and online health information seeking among *Never Users* and *Use but not for health* to inform and shape targeted and tailored interventions. Targeting interventions and educational materials to nonusers and users of the internet may improve internet and health literacy and support the ultimate goal of developing a more informed and empowered individual.

## Abbreviations

CHIS: California Health Interview Survey
eHealth: electronic health
NH: Non-Hispanic
OR: odds ratio
